# The Th17/Treg Immune Balance in Ulcerative Colitis Patients with Two Different Chinese Syndromes: Dampness-Heat in Large Intestine and Spleen and Kidney Yang Deficiency Syndrome

**DOI:** 10.1155/2015/264317

**Published:** 2015-03-01

**Authors:** Yang Gong, Lixing Liu, Xiaojuan He, Hongyan Zhao, Jing Yang, Li Li, Aiping Lu, Yifan Lin, Miao Jiang

**Affiliations:** ^1^The General Hospital of Shenyang Military Region, Shenyang, Liaoning 110840, China; ^2^Beijing Ditan Hospital, Capital Medical University, Beijing 100011, China; ^3^Institute of Basic Research in Clinical Medicine, China Academy of Chinese Medical Sciences, Beijing 100700, China; ^4^Institute of Basic Theory, China Academy of Chinese Medical Sciences, Beijing 100700, China

## Abstract

*Objective*. To investigate the Th17/Treg immune balance in the ulcerative colitis (UC) patients with two Chinese syndrome: dampness-heat in large intestine (DHLI) and spleen and kidney Yang deficiency (SKYD).* Methods*. Ninety UC patients (45 were diagnosed with DHLI and 45 with SKYD syndrome) and 23 healthy people were recruited. The serumIL-17 and TGF-*β*1 levels of these participants were measured with ELISA; the expression of IL-17 and TGF-*β* 1 in colonic mucosa tissue was determined with immunohistochemistry and the percentage of Th17 and Treg in peripheral blood with flow cytometry.* Results*. The levels of IL-17 and Th17 were significantly higher in both DHLI and SKYD groups than in healthy control group and higher in DHLI than in SKYD group (*P* < 0.05). The levels of TGF-*β*1 and Treg were significantly lower in the two UC patients groups than in healthy control group; and lower in SKYD group than in DHLI group (*P* < 0.05).* Conclusions*. UC with DHLI syndrome could be characterized by the elevation of Th17 and IL-17 levels, which indicated an accentuation of inflammatory reaction; UC with SKYD syndrome could be characterized by the reduction of serum Treg and TGF-*β*1 levels, which represented a depression of immune tolerance.

## 1. Introduction

Ulcerative colitis (UC) [1–3] is one of the commonly prevalent inflammatory bowel diseases (IBD) which represents a group of idiopathic, chronic, inflammatory intestinal conditions. UC is characterized by the inflammation of the intestine mucosa and submucosa, with a group of clinical symptoms of diarrhea, mucus purulent blood stool, and abdominal pain [4, 5]. Despite promising results with the use of biological therapy agents in the management of UC, currently there is no curative treatment for the long-term and recurrent disease [1, 6–8].

For a long time, UC has been regarded as mainly prevalent in the Western countries [9–11]; however, recently, a growing number of data showed that the incidence of this disease is significantly higher than before in Asia [12–14]. Chinese medicine (CM), as an essential part of the healthcare system in several Asian countries, has shown a promising clinical effect in the treatment of UC patients [15, 16]. Generally, a successful treatment for UC with Chinese medicine is based on the syndrome differentiation, which is one of the most important concepts in the practice of Chinese medicine theory.

In our previous study, the distribution of the major UC syndromes was analyzed using the clinical information from 198 UC patients [17]. In the 198 UC patients, 52.02% of population was diagnosed with dampness and heat in large intestinal syndrome, 25.76% with liver depression and spleen weakness syndrome, 22.22% with intestinal blood stasis syndrome, 21.72% with qi deficiency in spleen and kidney syndrome, 11.62% with Yang deficiency in spleen and kidney syndrome, 2.02% with yin and blood deficiency syndrome, and 2.53% with cold and dampness repression syndrome. Since DHLI was the most frequently seen syndrome in UC patients in clinical practice and the DHLI syndrome diagnosis is closely correlated with specific Chinese medicine therapy, it is necessary to explore the underlying mechanism of this syndrome and the difference between this syndrome and others.

From the viewpoint of clinical practice, DHLI syndrome was represented by a group of symptoms, such as mucus purulent blood stool, sensation of rectal tenesmus, anal burning pain, heat sensation, dry and bitter sensation in mouth, and yellow-thick or greasy coating; these symptoms showed opposite characteristics comparing with the symptoms indicating SKYD syndrome, including lingering dysentery, umbilical abdominal crymodynia, warmth and pressure relieved pain, soreness and weakness of waist and knees, cold sensation in body and four limbs, and deep and thin or weak pulse. The two syndromes DHLI and SKYD are reversely corresponding with regard to symptoms and signs; therefore, it can be assumed that there exists some underlying mechanism which showed opposite modulation tendency comparing these two syndromes. The assumption has been partially proved by some previous studies; in these studies, bioinformatics, genomics, and data mining techniques were used to decipher the mechanism of two syndromes which showed opposite clinical characters [19–21].

To date, although the pathogenesis of UC is still unclear, it is thought that this disease is closely related with abnormal immune function and the Th17/Treg immune balance which is also believed to play an important role in the pathogenesis process of UC [22, 23]. Th17 and Treg are originated from CD4^+^ T cells, existing in peripheral blood and spleen. Th17 (CD4^+^ IL-17^+^) cells are known as inflammatory helper T cells, important proinflammatory cells, promoting inflammation expanding and progressing with specific secretion of IL-17 [24]. Treg (CD4^+^ CD25^+^ Foxp3^+^) are suppressor cells with unique immune regulation, which mainly secrete transforming growth factor-beta (transforming growth factor-beta, TGF-beta), IL-10, and IL-4 cytokines. In the immune system, the normal level and function of Treg cells are one of the important regulation ways to maintain the body's immune tolerance, and the function of Treg cells can be achieved by inhibiting cytokines [25], which play an important role in the production and the proliferation of Treg cells and the mediation of immune tolerance [26, 27]. Th17 and Treg are closely related in the process of differentiation and transformation, while they also can be independent or unified in the body's immune response which forms an immune balance as a switch of a variety of autoimmune diseases. In short, Th17 is one of the immune promoting cells, while Treg is a kind of immune suppressing cell; thus, the two kinds of cells and their balance are closely related to the immune function. UC is regarded as a kind of chronic inflammatory disease characterized by an abnormal immunological response to microbial antigens in individuals. The abnormal immunological response includes both accentuation and underactivity of immunological response. According to CM theory, the immunological response accentuation often can be detected in UC patients with LIDH syndrome; conversely, underactivity of immunological response always happens in UC patients with SKYD syndrome. Thus, we assume that since the imbalance of Th17/Treg can be a reflection of the immunological response, it might be a guiding indicator of the differentiation of these two contrast CM syndromes.

Therefore, in this study, we designed a study to investigate the Th17/Treg immune balance in the UC patients with the Chinese syndromes of DHLI and SKYD, trying to explore underlying mechanism of these two syndromes of UC patients. The whole study was conducted according to the Declaration of Helsinki and the International Conference on Harmonization Tripartite Guideline on Good Clinical Practice [28]. Approvals from the appropriate research ethics committees were obtained before the trials began. All patients were asked to provide written, informed consent before participating.

## 2. Materials and Methods

### 2.1. Patients

**Figure 1:**
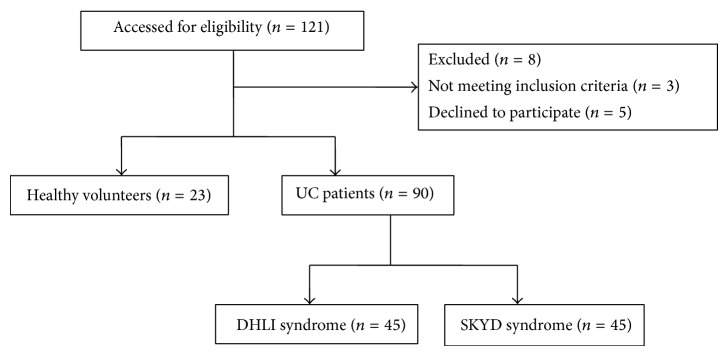
Flowchart for patient recruitment.

From July 1, 2010, to February 28, 2014, 90 in- and outward patients who were diagnosed with UC were recruited from the General Hospital of Shenyang Military Region; among these patients, 45 were diagnosed with the DHLI syndrome and 45 with SKYD syndrome. Twenty-three healthy volunteers were recruited from the community near the General Hospital of Shenyang Military.  Figure 1 shows the flowchart of the patient enrollment.

All UC patients enrolled were aged between 18 and 70 years, diagnosed with UC, the initial onset, chronic persistent, or chronic relapsing type, and diagnosed with DHLI or SKYD syndrome by two independent and experienced (with more than 5 years clinical experience) Chinese clinicians. Diagnostic facilities for high-quality endoscopy, radiology, and pathology should be available. The diagnosis of UC was made by clinical, laboratory, endoscopic, and histologic examinations in accordance with the suggested guidelines for the diagnosis and treatment of IBD, which were approved in China in 2007 [29].

DHLI syndrome and SKYD syndrome diagnosis was based on the Chinese consensus on diagnosis and treatment standard of inflammatory bowel disease. The details are quoted as follows [30, 31]:

3.1 Large intestine dampness-heat (LIDH) syndrome:

(1) Primary Symptoms. ① Mucus purulent blood stool; ② sensation of rectal tenesmus; ③ yellow-thick or greasy tongue coating; ④ slippery or soggy rapid pulse.

(2) Secondary Symptoms. ① Anal burning pain; ② heat sensation; ③ sagging or burning pain in the lower abdomen; ④ dry and bitter sensation in mouth with unpleasant smell; ⑤ short voiding of dark-colored urine.

(3) Diagnosis Criteria. The patient will be diagnosed with LIDH syndrome if he/she was diagnosed with: primary symptom ①, plus any other one of the primary symptoms (from ② to ⑤), plus any two of the secondary symptoms; orprimary symptom ①, plus any three of the secondary symptoms.

3.2 Spleen-kidney Yang deficiency (SKYD) syndrome:

(1) Primary Symptoms. ① Lingering dysentery with loose stool or undigested food in the stool; ② soreness and weakness of waist and knees; ③ cold sensation in body and four limbs; ④ poor appetite with reduced intake; ⑤ slightly enlarged tongue body or teeth print in tongue body, white and moist tongue coating; deep and thin or weak pulse.

(2) Secondary Symptoms. ① Diarrhea at dawn or before sunrise; ② umbilical abdominal crymodynia, relieved by warmth and pressure; ③ abdominal flatulence and borborygmus; ④ shortness of breath, no desire to speak; ⑤ pallor complexion.

(3) Diagnosis Criteria. The patient will be diagnosed with SKLD syndrome if he/she was diagnosed with: primary symptom ①, plus any other one of the primary symptoms (from ② to ⑤), plus any two of the secondary symptoms; or primary symptom ①, plus any three of the secondary symptoms.

Priority was given to the mild and moderate UC patients; cases with severe disease would also be enrolled if they did not need emergency therapy. None of the enrolled subjects had any serious complications, such as local stricture, intestinal obstruction, intestinal perforation, rectum polyp, toxic colonic dilatation, colon or rectum cancer, or anus diseases; patients who had received any CM treatment within 3 months were excluded. Women who were pregnant or preparing to be pregnant or lactating were excluded from the study.

### 2.2. Antibodies, Reagents, and Instruments

ELISA kits for the detection of IL-17 and TGF-*β*1 were obtained from the RB Co. Ltd., USA. The Varioskan Flash was purchased from Thermo Scientific, USA.

IL-17 and TGF-*β*1 antibodies for immunohistochemistry test were obtained from the Santa Cruz Biotechnology, Inc., USA. DAB kit was purchased from the ZSGB Bio, China. Olympus microscope (U-CMAD-2, konDS-FI2) was purchased from Olympus Corporation.

IL-17 PE, CD4 FITC, CD4 APC, PMA, ionomycin, CD25 APC, fixation/permeabilization buffer, and FOXP3 for the detection of Th17 and Treg in human blood by flow cytometry test were obtained from Becton, Dickinson and Company, USA. Canto II flow cytometer was purchased from Becton, Dickinson and Company.

### 2.3. Sample Collection and Preparation

Peripheral venous blood (8 milliliters in each subject) was collected from UC patients as well as healthy volunteers. The subjects had to be fasted for at least 12 hours before blood sample collection. The blood sample was kept in the room temperature for 1 h and then centrifugated for 10 minutes at 4°C, 3000 r/min. Supernatant was then frozen at −80°C.

The colon lesion tissue was collected from the UC patients and normal colon tissue from the healthy volunteers by endoscopy. After the fixation and paraffin embedding, the 4 *μ*m section of colon tissue of each subject was obtained for immunohistochemistry test.

### 2.4. Enzyme-Linked Immunosorbent Assay (ELISA)

ELISA assay was performed according to the manufacturer's instructions of the ELISA kits (RapidBio, USA) for IL-17 and TGF-*β*1. The OD value at 540 nm was measured. The concentrations of IL-17 and TGF-*β*1 were calculated according to the standard curve.

### 2.5. Immunohistochemistry Assay

The colon lesion tissue from the UC patients and the normal colon tissue from the healthy volunteers were collected by endoscopy. After the fixation and paraffin embedding, the sections (4 *μ*m) were incubated with rabbit polyclonal anti-TGF-*β*1 antibody or anti-IL-17 antibody (1 : 50 dilution; Santa Cruz Biotechnology, Inc.). After three washes in PBS, the sections were incubated with anti-rabbit IgG (1 : 300 dilution; Invitrogen). Finally, the sections were examined with DAB to show the positive products with brown-yellow color under the Olympus microscope (U-CMAD-2, konDS-FI2) and the data were analyzed by the software of Image-Pro Plus 6.0.

### 2.6. Flow Cytometric Analysis

For detecting the percentage of Th17 cells, the PBMCs were stimulated with 20 ng/mL phorbol 12-myristate-13-acetate and 1 *μ*g/mL ionomycin in the presence of 2 mmol/mL monensin (Sigma-Aldrich, USA) in 24-well plates. After being stimulated for 4 hours (37°C, 5% CO2), the cells were collected and washed once with PBS. The cells were then incubated with APC-CD3 antibody and PE-Cy5-CD4 antibody at 4°C for 30 minutes. Next, the cells were fixed and permeabilized and stained with anti-human PE-IL-17 antibody at 37°C for 25 minutes. For detecting the percentage of Treg cells, the PBMCs were washed in PBS. Then, the cells were stained with APC-CD3, PE-Cy5-CD4, PE-Cy7-CD8, and FITC-CD25 antibodies at 4°C for 30 minutes. Then, the cells were incubated with PE-Foxp3 antibody after fixation and permeabilization according to the manufacturer's instruction. All stained cells were analyzed by flow cytometry (FACSCalibur) and FlowJo software (Tristar, USA). The forward angle scattering light (FSC) and side scattering light (SSC) were adjusted to select the lymphocytes. Different cell subsets were detected by different cell labeling and gating. CellQuest software was used for data analysis and the percentage of positive cells was recorded.

### 2.7. Statistical Analysis

All data were analyzed by SPSS17.0 software. All data except demographic data were expressed as mean ± SD and were analyzed by means of Student's *t*-test or ANOVA followed by Fisher exact test for post hoc analysis. Demographic data was analyzed by means of chi-square test. A *P* value of less than 0.05 was considered statistically significant.

## 3. Results

### 3.1. Demographic Details and Clinical Features of UC Patients and Healthy Subjects

Among the 90 UC patients, 47 were male (52.2%), and the age was between 23 and 65 years (37.8 ± 8.8). In the healthy control group, there were 12 males in the 23 subjects (52.2%) with age ranging from 29 to 60 years (39.7 ± 8.4). There were no significant differences between the two groups regarding age and gender.

The clinical features of the DHLI syndrome and SKYD syndrome groups were demonstrated in Table 1. No difference was detected between the two groups in aspect of age, gender, biopsy type, and use of drugs including salicylazosulfapyridine (SASP), 5-aminosalicylic acid (5-ASA), traditional Chinese medicine (TCM), and integrative treatment (Chinese herb medicine plus salicylazosulfapyridine or 5-aminosalicylic acid).

There existed statistical differences between the DHLI syndrome and SKYD syndrome groups with regard to disease type, phase, duration, clinical activity index, and endoscopic index (*P* < 0.05). In DHLI syndrome group, more patients were diagnosed as the initial onset type compared to in SKYD group; accordingly, there were more patients with duration less than 5 years in DHLI group. There were also more patients in active phase and with higher disease index (disease activity index and endoscopic index) in DHLI group comparing with in SKYD group (*P* < 0.05).

### 3.2. Serum IL-17 and TGF-*β*1 Levels in Patients of Three Groups

Comparing with healthy control group, the level of serum IL-17 in both syndrome groups of UC patients increased significantly (*P* < 0.05); the level of serum TGF-*β*1 in the two UC groups decreased significantly (*P* < 0.05).

**Figure 2:**
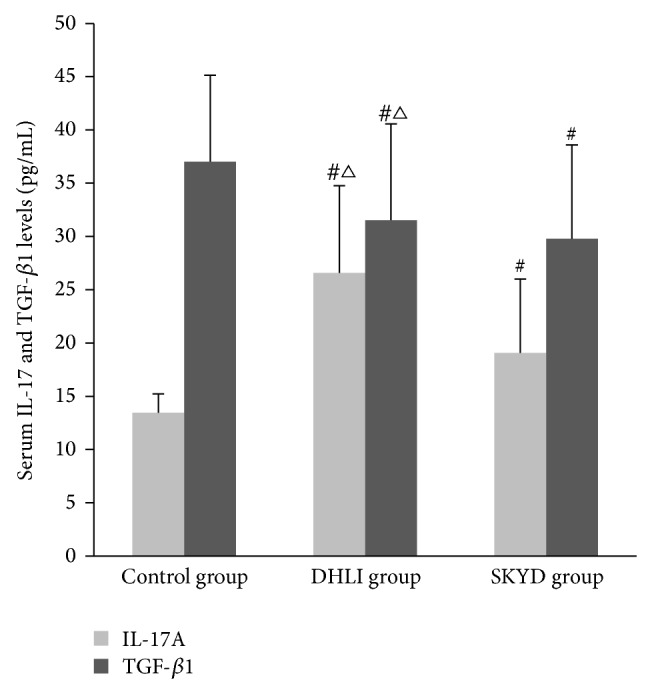
Serum IL-17 and TGF-*β*1 levels in patients of three groups (pg/mL). # indicated *P* < 0.05 comparing with control group; △ represented *P* < 0.05 comparing with SKYD group.

In DHLI group, IL-17 level was higher and TGF-*β*1 level was lower than in SKYD group (both *P* < 0.05), as shown in Figure 2 and Table 2.

### 3.3. The Expressions of IL-17 and TGF-*β*1 in Colonic Mucosa in Patients of Three Groups

**Figure 3:**
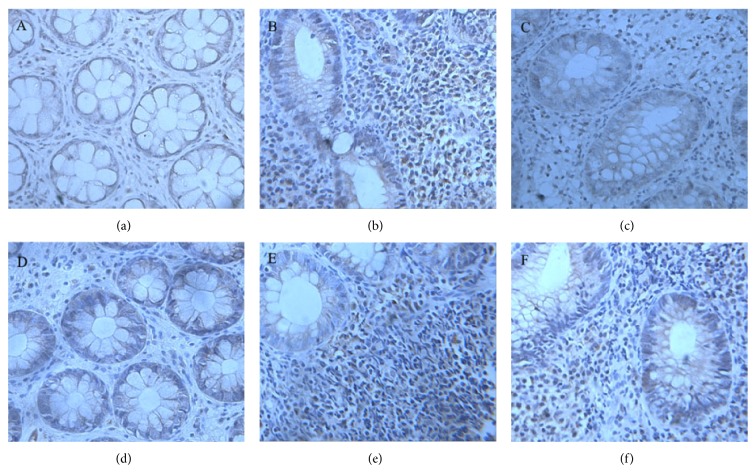
The expressions of IL-17 and TGF-*β*1 in colonic mucosa in three groups. (The expressions of IL-17 in colonic mucosa. (a) Control group; (b) dampness-heat in large intestine syndrome group (DHLI); (c) spleen and kidney Yang deficiency syndrome group (SKYD). The expressions of TGF-*β*1 in colonic mucosa. (d) Control group; (e) dampness-heat in large intestine syndrome group (DHLI); (f) spleen and kidney Yang deficiency syndrome group (SKYD).)

Compared with the healthy control group, the expression of IL-17 and TGF-*β*1 in colonic mucosa of two UC groups increased significantly (*P* < 0.05). In DHLI group, the expressions of IL-17 and TGF-*β*1 in colonic mucosa were significantly higher than in SKYD group (both *P* < 0.05) as shown in Table 2 and Figure 3.

### 3.4. The Percentages of Th17 and Treg in Peripheral Blood in Three Groups

**Figure 4:**
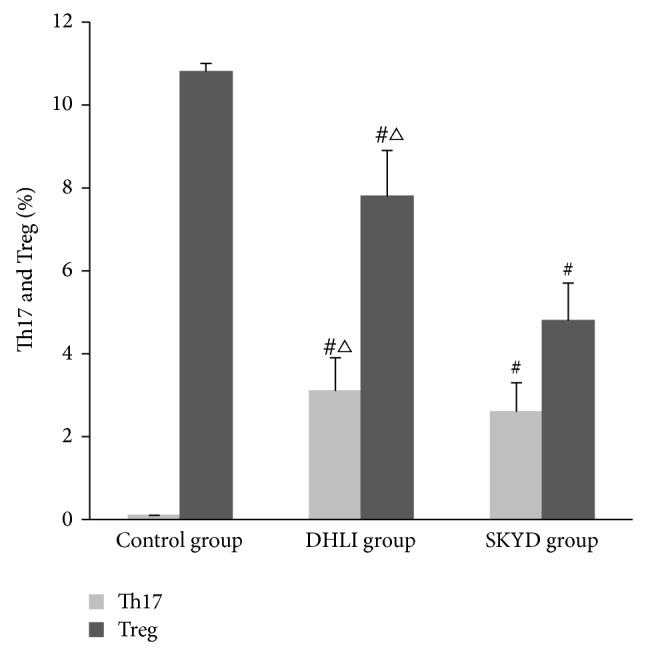
The percentages of Th17 and Treg in UC patients and healthy people (%) (^#^ *P* < 0.05 versus control group; ^△^ *P* < 0.05 versus spleen and kidney Yang deficiency syndrome group).

**Figure 5:**
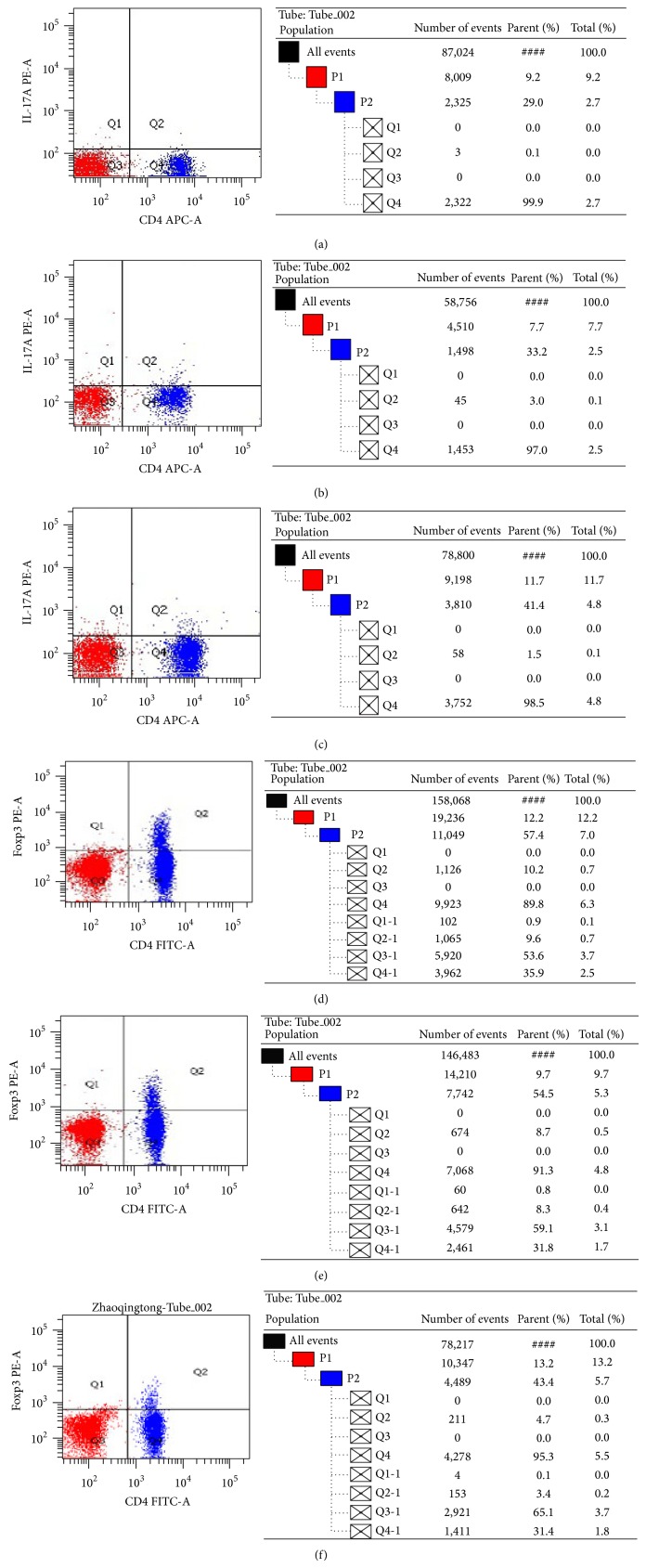
Flow cytometric analysis of Th17 cells and Treg cells in the peripheral blood of three groups. (The percentages of Th17 in UC patients and healthy people (%). (a) Control group; (b) dampness-heat in large intestine syndrome group; (c) spleen and kidney Yang deficiency syndrome group. The percentages of Treg in UC patients and healthy people (%). (d) Control group; (e) dampness-heat in large intestine syndrome group; (f) spleen and kidney Yang deficiency syndrome group.)

Comparing with the healthy control group, the percentage of Th17 in peripheral blood increased significantly in two UC groups (*P* < 0.05), while the percentage of Treg decreased significantly (*P* < 0.05). In DHLI group, the percentage of Th17 was significantly higher than in SKYD group (*P* < 0.05); the percentage of Treg was significantly lower than in SKYD group (*P* < 0.05) as shown in Table 2 and Figures 4 and 5.

## 4. Discussion

To date, it has been fully recognized that the Th17/Treg imbalance is closely related to development of UC, and many efforts have been made in this field in order to explore the exact impact of Th17/Treg imbalance on UC [32–34]. However, none of the studies paid attention to the difference between one subgroup and another subgroup of UC patients, which might be the key point for successful treatment selection. This is the first study to discuss the differentiation of two common types of CM diagnosis (DHLI and SKYD syndromes) in UC patients in the context of Th17/Treg balance system. The outcomes might provide some evidence for the subgrouping of UC patients based on CM theory to enlighten the better treatment and further understanding of the biological mechanism of UC patients with different CM syndrome.

In Chinese medicine theory, syndrome, which is also called zheng or pattern, is the basic unit and a key concept. Syndrome is the abstraction of a major disharmonious pathogenesis, which is identified from a comprehensive analysis of clinical information from four main diagnostic TCM methods: observation, listening, questioning, and pulse analyses [35]. In brief, all diagnostic and therapeutic methods in Chinese medicine are based on the differentiation of Chinese medicine syndrome, a concept that has been used in China for over 3,000 years [36, 37]. Syndrome can be understood as a guideline for patient classification in clinical practice from an alternate viewpoint/dimension compared to a biomedical disease diagnosis. For example, patients suffering from the same disease might be classified with different syndrome, whereas different diseases might be categorized with the same syndrome. Different syndrome may occur for one patient at the same time, and syndrome classification is dynamic because syndrome can change during the evolution of a disease. Thus, syndrome classification could be considered to be a further stratification in patients with a single disease, allowing clinicians to obtain more accurate patient classifications. At present, a syndrome diagnosis is integrated with a biomedical diagnosis in clinical practice, and integrative medicine emerges as an optimal approach for achieving higher efficacy [35].

DHLI syndrome is the most common syndrome among UC patients in clinical practice according to our previous study; it covers more than a half of UC patients [17]. Clinically, UC patients with DHLI syndrome show a group of symptoms such as mucus purulent blood stool, accompanied with sensation of rectal tenesmus, anal burning pain, heat sensation, dry and bitter sensation in mouth, and yellow-thick or greasy coating in the UC patients with dampness-heat in large intestine syndrome, while UC patients with SKYD syndrome often manifest different or opposite symptoms which are mainly lingering dysentery, together with umbilical abdominal crymodynia, warmth and pressure relieved pain, soreness and weakness of waist and knees, cold sensation in body and four limbs, and deep and thin or weak pulse. Given the opposite characteristics of the DHLI syndrome and SKYD syndrome, in our study design, we selected these two syndromes and tried to compare their immune imbalance features in order to explore the underlying mechanism of the classification of the two syndromes of UC patients.

Our results indicated that, comparing with health subjects, UC patients showed concurrent changes in the levels of IL-17 and TGF-*β*1 and the percentage of Th17 and Treg, which implied the exacerbation of inflammation in UC patients. The increased expression of TGF-*β*1 in colonic mucosa might be due to the redistribution of TGF-*β*1 after the UC onset and was relatively concentrated in the colonic mucosa. These results were accordant with previous studies [38].

However, between the DHLI and SKYD groups, there exists significant difference in the Th17/Treg imbalance. In UC patients with DHLI syndrome, most cases were diagnosed as the initial onset type and in the active phase, comparing with those with SKYD syndrome. More notably, UC patients with DHLI syndrome showed more severe inflammation condition, with shorter clinical course and higher clinical activity index and endoscopic index, while the cases with SKYD syndrome could be characterized by relatively milder inflammation and longer clinical course. These results indicated that there were significant different changes in immune system in patients with different Chinese medicine syndromes although they were diagnosed with the same disease, and, consequently, these patients with different syndrome diagnosis might accept different therapy for achieving better effect.

There are still some limitations in this study. Since there are more than two types of syndromes in UC patients, our study could not cover all syndromes of UC patients; we selected only the two most commonly seen syndromes. More studies are still warranted to explore the underlying mechanism on syndrome classification of UC patients.

## 5. Conclusion

UC with DHLI syndrome could be characterized by the elevation of Th17 and IL-17 levels, which indicated an accentuation of inflammatory reaction; UC with SKYD syndrome could be characterized by the reduction of serum Treg and TGF-*β*1 levels, which represented a depression of immune tolerance. The different mechanism on immune imbalance of UC patients might provide new insights for individualization of treatment in the future.

## Figures and Tables

**Table 1:** Clinical data in DHLI syndrome and SKYD syndrome groups ^*^.

Characteristics	DHLI group (*n* = 45)	SKYD group (*n* = 45)	*P* value
Male	26 (57.8)	21 (45.7)	0.291
Age-year	39.0 ± 8.6	36.6 ± 8.9	0.192
Medication history			
SASP	5 (11.11)	6 (13.33)	1.000
5-ASA	19 (42.22)	21 (46.67)	0.416
TCM	11 (24.45)	7 (15.56)	0.430
Integrative treatment	10 (22.22)	11 (24.45)	1.000
Disease type			
Initial onset type^#^	13 (28.9)	2 (3.4)	0.002
Chronic persistent type	15 (33.3)	19 (42.2)	0.384
Chronic relapsing type	17 (37.8)	24 (53.3)	0.138
Active phase^#^	31 (68.9)	19 (42.2)	0.011
Remission phase^#^	14 (31.1)	26 (57.7)	0.011
Disease level			
Mild	12 (26.7)	16 (35.6)	0.362
Middle	29 (64.5)	28 (62.2)	0.827
Severe	4 (6.9)	1 (2.2)	0.167
Duration of disease			
*⩽*60 months^#^	19 (42.2)	10 (22.2)	0.042
>60 months^#^	26 (57.8)	35 (77.8)	0.042
Clinical activity index^#^	7.4 ± 2.3	6.4 ± 2.1	0.036
Endoscopic index^#^	6.0 ± 2.2	4.8 ± 1.7	0.010
Biopsy type^☆^			
0	8 (17.8)	11 (24.4)	0.438
1	10 (22.2)	8 (17.8)	0.598
2	12 (26.7)	13 (28.9)	0.814
3	8 (17.8)	7 (15.6)	0.777
4	3 (6.7)	4 (8.9)	0.694
5	4 (8.9)	2 (4.4)	0.398

^*^Data was presented as case number (%). Age, clinical activity index, and endoscopic index were displayed as mean ± SD.

^#^ *P* < 0.05. There was significant difference between the DHLI and SKYD groups.

^☆^0, structural change only; 1, chronic inflammation; 2, lamina propria neutrophils; 3, neutrophils in epithelium; 4, crypt destruction; and 5, erosions or ulcers [18].

**Table 2:** Levels of IL-17 and TGF-*β*1 and percentages of Th17 and Treg in DHLI, SKYD, and healthy control groups (mean ± SD) ^*^.

	DHLI	SKYD	Healthy control
Serum level (pg/mL)			
IL-17	26.58 ± 8.19^#△^	19.09 ± 6.90^#^	13.46 ± 1.77
TGF-*β*1	31.53 ± 9.04^#△^	29.80 ± 8.79^#^	37.01 ± 8.11

Expression level in colonic mucosa (OD value)			
IL-17	0.28 ± 0.11^#△^	0.23 ± 0.09^#^	0.09 ± 0.02
TGF-*β*1	0.0052 ± 0.0013^#△^	0.0045 ± 0.0008^#^	0.0025 ± 0.0005

Percentages of Th17 and Treg in peripheral blood (%)			
Th17	3.1 ± 0.8^#△^	2.6 ± 0.7^#^	0.1 ± 0.0
Treg	7.8 ± 1.1^#△^	4.8 ± 0.9^#^	10.8 ± 0.2

^*^indicated *P* < 0.05 comparing with control group; ^△^represented *P* < 0.05 comparing with SKYD group.
